# Unraveling the Gut-Brain Axis in Multiple Sclerosis: Exploring Dysbiosis, Oxidative Stress, and Therapeutic Insights

**DOI:** 10.7759/cureus.47058

**Published:** 2023-10-15

**Authors:** Mouhammad Sharifa, Tanmay Ghosh, Omar A Daher, Pramod Bhusal, Yasoob A Alaameri, Javeria Naz, Chukwuyem Ekhator, Sophia B Bellegarde, Pakeezah Bisharat, Viralkumar Vaghani, Azlaan Hussain

**Affiliations:** 1 Medicine, University of Aleppo, Aleppo, SYR; 2 Medical Education, Dinabandhu Andrews College, West Bengal, IND; 3 Obstetrics and Gynaecology, Beirut Arab University, Tripoli, LBN; 4 Internal Medicine, College Of Medical Sciences, Bharatpur, NPL; 5 Neurosurgery, Baghdad Medical City Complex, Baghdad, IRQ; 6 Internal Medicine, Jinnah Sindh Medical University, Karachi, PAK; 7 Neuro-Oncology, New York Institute of Technology, College of Osteopathic Medicine, Old Westbury, USA; 8 Pathology and Laboratory Medicine, American University of Antigua, St. John's, ATG; 9 Internal Medicine, Khyber Medical University, Peshawar , PAK; 10 Biomedical Informatics, The University of Texas Health Science Center, Houston, USA; 11 Medicine and Surgery, Mayo Hospital, Lahore, PAK

**Keywords:** probiotics, fecal microbiota transplantation, environmental factors, therapeutic interventions, inflammasome, oxidative stress, dysbiosis, gut microbiota, gut-brain axis, s: multiple sclerosis

## Abstract

This comprehensive review delves into the intricate relationship between the gut microbiota and multiple sclerosis (MS), shedding light on the potential therapeutic avenues for this complex autoimmune disease. It emphasizes the multifactorial nature of MS, including genetic, environmental, and gender-related factors. Furthermore, the article highlights the emerging role of gut microbiota in MS pathophysiology, particularly in terms of gut dysbiosis, oxidative stress, and inflammasome activation within the gut-brain axis. This interplay raises intriguing questions about how the gut microbiota influences the onset and progression of MS. Environmental factors, such as diet and pollutants, add further layers of complexity to the connection between gut health and MS risk. This review also discusses promising therapeutic interventions, such as fecal microbiota transplantation, probiotics, dietary adjustments, and gut-derived metabolites that offer potential avenues for managing MS. It underscores the need for ongoing research to fully unravel the complexities of the role of the gut-brain axis in MS. Ultimately, this article provides a comprehensive exploration of the topic, offering hope for novel preventive and therapeutic strategies that could significantly improve the lives of individuals affected by this challenging autoimmune condition.

## Introduction and background

Multiple sclerosis (MS) is a complex autoimmune disease that predominantly affects young adults, causing damage to the central nervous system, specifically the brain and spinal cord. MS presents enormous challenges to healthcare and society, with more than one million current cases in Europe and rising numbers globally [1]. Although its exact cause is unknown, it is thought to be the consequence of a confluence of genetic, environmental, and hormonal variables, with women being more at risk [2]. The prevalence of MS is also influenced by geographic variables, race, and ethnicity [3,4]. Emerging research has revealed the involvement of gut microbiota in the pathophysiology of MS, offering a fresh viewpoint on the gut-brain axis, although, the precise interaction of these components is still unknown. It is now understood that the gut microbiome, a diverse community of bacteria found in the gastrointestinal tract, plays a crucial role in regulating the immune response, protecting the body from infections, and preserving general body homeostasis [5]. However, alterations in the immune system's response and increased systemic inflammation, particularly in autoimmune illnesses, have been linked to disruptions in the delicate balance between the host and its microbiota [6]. Oxidative stress is a major factor in MS, contributing to neurodegeneration, demyelination, and chronic inflammation. Although the precise processes are still unclear, recent studies have linked the course of autoimmune illnesses like MS to inflammasome activation, a response of the innate immune system [7]. In this intricate interplay between gut dysbiosis, oxidative stress, and inflammasome activation in MS, the gut-brain axis, a bidirectional communication mechanism linking the gut and the brain, appears to be key [8,9]. This review article focuses on exploring the interplay of the gut microbiota-brain axis in identifying potential therapeutic options for MS.

According to recent studies, gut dysbiosis, or an imbalance in the makeup of the gut microbiota, might affect redox signaling and hasten the onset of neurological diseases via the gut-brain axis [9,10]. This raises concerns regarding the gut-brain axis' possible participation in demyelination and neurodegeneration in MS. Investigations are being done to determine how gut dysbiosis, gut microbiota metabolites, and their effects on MS onset and progression are related. Understanding how the gut-brain axis controls this process in the setting of MS is a crucial field of study because oxidative stress is a recognized cause of neuroinflammation. Epidemiological statistics also demonstrate the important influence of environmental variables on autoimmune disorders. This further connects the environment, gut health, and the risk of developing MS by taking into account the role external influences play in altering gut-related metabolites. Examining these connections can offer important insights into potential prevention strategies.

Globally, and especially in low- and middle-income nations, there has been an increase in the prevalence of neurological and metabolic illnesses, including MS, which is mostly linked to environmental changes. The gut microbiota is an intriguing area for study into disease susceptibility and therapeutic approaches because of its complicated role in the gut-brain axis. Although practical use requires accuracy and more research, manipulating the microbiota through techniques like fecal microbiota transplantation (FMT) and probiotics provides promising therapy for MS [11,12]. An increasing amount of investigation is being done on dietary variables, with diets high in antioxidants and low in inflammatory foods showing promise in the treatment of MS [13,14]. Research is ongoing to better understand how nutrition affects the gut-brain axis and how that affects MS development, as well as to examine the possibility of dietary treatments as therapeutic strategies. In conclusion, the complex interactions between gut dysbiosis, oxidative stress, inflammasome activation, and environmental variables within the framework of the gut-brain axis provide a great deal of promise for explaining the riddles of MS. For those who suffer from this crippling autoimmune ailment, research in this area strives to give fresh perspectives on disease processes, prevention, and cutting-edge therapy approaches, giving hope for improved outcomes and quality of life.

## Review

Pathogenesis

The central nervous system (CNS) is affected by inflammation, demyelination, axonal damage, and axonal loss in multiple sclerosis (MS), a complicated neurological condition. Although the exact etiology of MS is still unknown, it is commonly accepted that a mix of genetic, environmental, and immunological factors is to blame. Inflammation of the central nervous system is the main factor in the development of MS. The main cause of damage in MS patients is this inflammatory response. Investigations are being done to determine the precise factors that cause this inflammation. Genetics, environmental factors, and infectious organisms are thought to play important roles in the onset of MS [15]. The experimental autoimmune encephalomyelitis (EAE) model has been used by researchers to better understand the immunological mechanisms causing MS. Although this model has given us useful information, it's important to recognize the fundamental contrasts between EAE and real MS as well as the complex structure of MS itself [16].

Understanding the differences between innate and adaptive immune responses is essential for comprehending the etiology of MS. Microbial compounds trigger the innate immune response by activating certain receptors, notably toll-like receptors (TLRs), in an unspecific way. The pathogen-associated chemicals that are specific to various kinds of pathogens are recognized by these TLRs. The production of cytokines that control the adaptive immune response results from the activation of certain TLRs [17]. When TLRs are engaged, dendritic cells (DCs) have a significant impact on the immunological response. Regulatory T cells are stimulated by semi-mature DCs to release inhibitory cytokines like TGF-β or IL-10 [18]. DCs polarize CD4+ T cells into different phenotypes, such as Th1, Th2, and Th17, as they continue to develop. Inflammation, a defining feature of MS, is encouraged by the Th1 and Th17 phenotypes.

The adaptive immune response, on the other hand, is initiated when specific antigens are presented to T lymphocytes by antigen-presenting cells (APCs), which include B cells, dendritic cells, microglia, and macrophages. One of the most important steps at the beginning of the adaptive immune response is the interaction between APCs and T cells. In response to certain interleukins, CD4+ T cells can develop into Th1, Th2, and Th17 effector cells. Th2 cells release anti-inflammatory cytokines like IL-4 and IL-13, whereas Th1 cells emit proinflammatory cytokines like interferon-gamma. In MS, Th17 cells, a recently identified subgroup, release IL-17 and encourage inflammation. These effector T cells go to the brain, where they aid in demyelination and axonal degeneration [17].

Another essential CD4+ T cell type in the pathophysiology of MS is Regulatory T cells (T reg). T reg cell counts are comparable between MS patients and controls, however, MS patients have a smaller functional T reg cell population [19]. Interferon beta (IFN-β) has been demonstrated to improve CD4+ regulatory T-cell activity, providing a possible therapeutic route [20]. In addition to CD4+ T cells, CD8+ T cells also contribute to the pathophysiology of MS. Through cytotoxic processes, CD8+ cells can prevent CD4+ T cells from proliferating, which renders them inactive. Furthermore, CD8+ T lymphocytes can destroy glial cells, exposing axons and causing axonal injury [18]. The pathogenesis of MS is also influenced by B cells and the byproducts they produce. In the cerebral fluid of MS patients, oligoclonal bands, which indicate polyclonal antibodies, are a recognized diagnostic characteristic. Although their precise target is uncertain, these antibodies target particular antigens. Further complicating their involvement in MS is the fact that B cells may generate both pro-inflammatory and anti-inflammatory cytokines [21,22].

MS susceptibility is greatly influenced by genetic factors. There is a significant hereditary component to MS, according to studies of families and twins, with first-degree relatives of MS patients showing a 40-fold greater vulnerability. The DR antigen-containing human leucocyte antigen (HLA) locus on chromosome 6p21 has been connected to MS vulnerability. On several chromosomes, additional susceptibility loci have been discovered [23]. Environmental elements, such as exposure to infectious agents and vitamin D/sunlight levels, are also very important in determining the likelihood of developing MS. Before the age of 15, moving from one place to another might change a person's chance of developing MS, indicating that environmental variables are at work [24]. Epstein-Barr virus, Mycoplasma pneumonia, and human herpes virus type 6 (HHV6) have all been investigated for their potential involvement in MS, with molecular mimicry being a plausible theory [25]. Moreover, sunlight exposure and adequate vitamin D levels have been associated with a protective effect [26].

In conclusion, complex immunological mechanisms, environmental triggers, and genetic predisposition interact in a variety of ways throughout the development of multiple sclerosis. The main cause of tissue damage is inflammation inside the central nervous system, and several immune cell subsets, such as CD4+ and CD8+ T cells, B cells, and regulatory T cells, are essential to the course of the disease. An individual's vulnerability to MS is further influenced by genetic and environmental variables. For the creation of specific treatments and better management of this crippling neurological illness, a deeper understanding of these intricate interconnections is crucial.

Gut-brain axis: the microbiota connection

The intricate relationship between the gut and the brain has fascinated scientists for centuries. The first verified account of a probable link between the gut and the brain didn't appear until the 19th century. William Beaumont, a physician for the US Army, made this discovery while tending to Alexis St. Martin, a Canadian fur trader who had been accidentally shot at close range. A remarkable phenomenon was seen by Beaumont throughout the course of the therapy: St. Martin's emotional state, particularly when he was agitated or furious, had a considerable influence on the rate of his digestion. The understanding of the gut-brain axis was first developed as a result of this coincidental discovery, which suggested a strong connection between the brain and the gut [27,28].

Since that crucial period in the 19th century, considerable advancements have been made in the study of neurogastroenterology. It has grown beyond anecdotal findings to incorporate sophisticated brain imaging techniques and animal research models. These developments have clarified the microbiota-gut-brain axis (MGBA), which Collins and Bercik (2009) describe as the intricate and reciprocal connection between the gut and the brain [29]. Most neurotransmitters produced in the gut cannot go directly from the gut to the brain, although they can affect the enteric nervous system (ENS). Brain activity and behavior are eventually impacted by this indirect influence on the ENS. Additionally, the creation of enzymes that control the metabolism of tryptophan allows the gut microbiota to control the availability of tryptophan, the precursor of serotonin, in the brain [30].

The function of the gut microbiota goes much beyond just digesting. It plays a key role in regulating the gut-brain axis, which has an impact on a variety of elements of human health and illness. A variety of health issues can result from dysbiosis, which is defined as an imbalance or disruption in the makeup of the gut microbiota. The following are some effects of dysbiosis: (1) Enhanced cellular degeneration: cellular degeneration can be brought on by dysbiosis, which can send signals to the brain. According to Noble et al. (2017), this cellular damage may speed up the development of neurodegenerative disorders [31]. (2) Low-grade inflammation: dysbiosis and low-grade inflammation are frequently seen together in the body. Chronic inflammation has the potential to aggravate already-existing neurological diseases as well as play a role in the emergence of new ones [32,33]. (3) Increased oxidative stress: the imbalances in the gut microbiota might encourage oxidative stress and worsen these disorders [34]. (4) Mood and behavioral disorders: The relationship between MGBA research and mood and behavioral disorders is possibly one of the most fascinating elements of the field. Dysbiosis has been connected to illnesses such as autistic spectrum disorders, depression, and anxiety [35,36].

Developing new treatment strategies will be significantly impacted by understanding the MGBA. Modifying the composition and activity of the gut microbiota by targeting it may open up new therapeutic possibilities for a variety of neuropsychiatric, neurological, and neurodevelopmental diseases. However, it's crucial to understand that the MGBA is only one component of the intricate picture of brain-gut connections, and further study is required to realize its full potential.

The gut microbiota-brain axis in MS

Trillions of commensal bacteria reside in the gastrointestinal system and are essential for maintaining the integrity of the gut barrier and balancing pathogenic and helpful bacteria [37]. For general health, it is important to maintain this precise balance. Gut dysbiosis is a disorder that develops when this equilibrium is upset. In the case of MS, gut dysbiosis is a significant factor in the development of this disease. The formation of reactive oxygen species (ROS) by the gut microbiota is one of the main mechanisms influencing the relationship between gut dysbiosis and MS. Both pathogenic and commensal bacteria can modify their mitochondrial activity, which can produce ROS either directly or indirectly by producing active metabolites such as short-chain fatty acids (SCFA) and formyl-peptides [38]. Excessive ROS generation, which is frequently brought on by pathogenic bacteria, can harm intracellular signaling pathways and worsen inflammation in MS patients [39].

ROS has a variety of functions in the immune system. They play a crucial role in the innate immune system's activation of the inflammasome. Inflammation and, occasionally, inflammatory-mediated cell death (pyroptosis) can result from the ROS-stimulated production of proinflammatory cytokines. The pathogenic developments in MS may be exacerbated by this inflammatory cascade [39]. Complex interactions exist between ROS and the gut microbiome. While pathogenic bacteria encourage oxidative stress, certain commensal bacteria have antioxidant and anti-inflammatory effects [39]. The significance of preserving a balanced gut microbiota for immune system modulation is highlighted by this disparity.

The nuclear factor erythroid 2-related factor (Nrf2) is a crucial regulator in the fight against oxidative stress. An essential part of cellular antioxidative defense processes and the response to different stressors is played by the transcription factor Nrf2 [40]. Nrf2 protects against elevated ROS levels and neuroinflammation in cases of MS. Through their metabolites, some bacteria in the gut, including Lactobacillus rhamnossus and Clostridium spp., can reduce inflammation brought on by the nuclear factor kappa B (NF-kB) pathway [41,42]. Research is still being done on the complex interplay between these pathways in gut health and how it affects MS.

Multiprotein complexes called inflammasomes are essential for immunological responses. The NLRP3 inflammasome has received interest concerning MS [43]. Through the production of proinflammatory cytokines, activation of the NLRP3 inflammasome can cause inflammation. Inflammasome-related gene variations have been linked to MS in genomic research. However, there is an ongoing study into the precise function of inflammasomes in the pathophysiology of MS. It is thought that inflammation brought on by oxidative stress in MS is facilitated by mitochondrial ROS and oxidized mitochondrial DNA activating the NLRP3 inflammasome [44,45].

The sum of all environmental exposures a person has over the course of a lifetime is referred to as the exposome [46]. It includes both internal and external exposomes, such as metabolism, gut flora, and inflammation. Examples of external exposomes include environmental pollutants and lifestyle variables. Deciphering the intricate interactions between genetics, environment, and MS susceptibility requires an understanding of the exposome. Exposomes can affect oxidative stress and redox signaling in MS [46,47]. Increased ROS production and gut dysbiosis can be brought on by things like pesticide exposure, exposure to environmental contaminants, and dietary changes. For instance, long-term exposure to pesticides can alter the gut flora and increase oxidative stress through a variety of methods [48,49]. Antioxidant and anti-inflammatory active compounds are often produced by gut microbes. Tryptamine is a neuromodulator with antioxidant characteristics that is produced when commensal bacteria break down tryptophan. Deficits in tryptophan-derived neurotransmitters like serotonin and melatonin, which are both essential for sleep, are frequently linked to MS comorbidities, including depression [50,51]. By lowering oxidative stress, indoles, another byproduct of tryptophan metabolism, have shown neuroprotective benefits [52].

Therapeutic interventions targeting the gut microbiota-brain axis in MS

Various therapeutic interventions have been explored to target the gut microbiota-brain axis in MS. These interventions encompass antibiotics, probiotics, gut-derived metabolites, fecal microbiota transplantation (FMT), and dietary modifications. Experimental autoimmune encephalomyelitis (EAE) can be treated with antibiotics to reduce symptoms similar to MS, according to animal research [53-55]. A change in the gut flora caused by broad-spectrum antibiotics lessens the severity of EAE in a way that depends on T reg [55]. This impact is unique to the use of oral antibiotics, highlighting the significance of gut microbial regulation. It has been shown that antibiotics administered before EAE induction or during presymptomatic phases can guard against the condition [56]. However, using antibiotics while having clinical EAE might make symptoms worse. The effects of antibiotics on MS in human research are limited, with just a few small trials suggesting possible advantages. Doxycycline combined with IFN-β reduced relapse rates, improved disability measures, and reduced gadolinium-enhancing lesions [57,58]. Minocycline delayed conversion from clinically isolated syndrome to MS over six months [59].

Probiotics have drawn attention as possible treatments for MS. In rodent EAE models, various probiotics, primarily Lactobacillus strains, have shown promise in ameliorating disease symptoms [60,61]. However, the results are inconsistent and often contradictory. Clinical trials in people with MS are scarce, but small studies using probiotic mixes have reported improvements in disability scores, depression, anxiety, and inflammatory markers [62,63]. People with MS frequently use probiotic supplements, but reliable clinical studies are required to prove their effectiveness. The therapeutic potential of gut-derived metabolites, in particular SCFAs, has been studied. To lessen the severity and inflammation of EAE, increasing SCFA levels through high-fiber diets or direct supplementation has shown potential [64]. Particularly propionate supplementation has shown immunomodulatory effects in people, lowering Th17 cells and raising T reg. In MS patients, long-term propionate supplementation lowered relapse rates, stabilized disability, and stopped brain shrinkage [65]. Tauroursodeoxycholic acid (TUDCA), bile acids, and other gut-derived metabolites have also been investigated and have shown promise in controlling neuroinflammation [66].

FMT has also been suggested as a way to help MS patients regain healthy gut flora. Potential advantages include decreased microglia and astrocyte activation, enhanced blood brain barrier (BBB) integrity, and clinical stability, according to studies in EAE models and a case study involving a patient with MS [67,68]. However, clinical trials investigating FMT in MS are ongoing, and challenges related to donor selection, processing, administration route, and recipient premedication need to be addressed [69]. Manipulating calorie intake and diet composition can modulate CNS autoimmunity through changes in the gut microbiota. Dietary restriction (DR), ketogenic diets, high-fiber diets, and certain dietary adjustments have all been studied in MS models and patients [70,71]. DR has demonstrated anti-inflammatory benefits and enhanced gut flora richness. It is defined by calorie restriction without starvation. As mentioned earlier, high-fiber diets can encourage the creation of SCFA, and several therapeutic trials are looking into how they can affect MS patients [64]. Other dietary strategies, such as methionine restriction and tryptophan-free diets, have shown promise in easing the severity of EAE. Although there is little study in this area, therapies like phosphatidylcholine supplements that target gut epithelial integrity have shown promise in treating other inflammatory illnesses [72]. This strategy needs more investigation in MS.

The gut microbiota may be impacted by disease-modifying therapies (DMTs) and immunosuppressive medications used in MS treatment. Some treatments, including fingolimod, dimethyl fumarate, interferon-beta, and glatiramer acetate, have been linked to changes in the makeup of the gut microbiota [73-76]. The gut microbiome is impacted by alemtuzumab's induction of systemic lymphocyte depletion [77,78]. Steroids, which are frequently used to control relapses, might alter the makeup of the gut flora [79]. Antidepressants and cannabis, among other symptomatic medications, may have an impact on the makeup of the gut flora [80,81].

## Conclusions

Investigating the gut microbiota-brain axis in multiple sclerosis uncovers a complex interplay of factors with the potential to enhance our understanding of this autoimmune disease. Since MS affects millions of people globally, novel strategies must be developed. This review paper emphasizes the importance of the gut-brain axis in MS and describes how gut dysbiosis affects oxidative stress, inflammation, and the immune system. Environmental elements, such as dietary habits and contaminants, connect environmental stressors, gut health, and MS risk, providing new opportunities for preventive measures. Although standardization and long-term effects remain challenges, therapeutic treatments like probiotics and fecal transplantation show promise for the therapy of MS. Biomarker research, including microbial and metabolomic signatures, offers hope for personalized MS diagnosis and treatment. This research offers optimism for improved outcomes, quality of life, and groundbreaking therapies in the battle against MS, with the gut microbiota-brain axis playing a pivotal role.
